# The complete mitochondrial genomes and phylogenetic analysis of two Chinese endemic cave fishes, *Sinocyclocheilus guilinensis* and *S. huangtianensis* (Cypriniformes: Cyprinidae)

**DOI:** 10.1080/23802359.2025.2460776

**Published:** 2025-02-02

**Authors:** Guojin Yao, You He, Chenhong Li

**Affiliations:** a Shanghai Universities Key Laboratory of Marine Animal Taxonomy and Evolution, Shanghai Ocean University, Shanghai, China; b Engineering Research Center of Environmental DNA and Ecological Water Health Assessment, Shanghai Ocean University, Shanghai, China; c Shanghai Synchrotron Radiation Facility, Shanghai Advanced Research Institute, Chinese Academy of Sciences, Shanghai, China

**Keywords:** *Sinocyclocheilus guilinensis*, *S. huangtianensis*, mitochondrial genome, molecular phylogeny, cave fishes

## Abstract

*Sinocyclocheilus* is a group of cyprinid fishes endemic to China distributed in the karst region of southwest China. In this study, the complete mitogenomes of *Sinocyclocheilus guilinensis* Ji, 1985 and *Sinocyclocheilus huangtianensis* Zhu et al. 2011 are reported and characterized. Both genomes contain 13 protein-coding genes, 22 tRNAs, 2 rRNAs, and a non-coding control region, with lengths of 16,576 bp and 16,578 bp, respectively. Phylogenetic analysis shows that *S. guilinensis* is the earliest branching species in the *S. jii* group, while *S. huangtianensis* and *S. jii* are sister groups. These mitochondrial genomes are valuable for studying systematics of *Sinocyclocheilus*.

## Introduction

*Sinocyclocheilus* is a group of cave fish endemic to China, belonging to the order Cypriniformes, family Cyprinidae, with about 78 species up to date (Jiang et al. 2023; Xu et al. 2023). It is mainly distributed in the Yunnan-Guizhou plateau where karst landscapes are richly developed, including eastern Yunnan, southern Guizhou, and western and northwestern Guangxi (Zhao and Zhang 2006). However, a few species such as *S. grahami*, *S. wumengshanensis* and *S. huizeensis* are distributed in the Jinshajiang River drainage (Chu and Cui 1985; Li et al. 2003; Cheng et al. 2015) and *S. multipunctatus* has been recorded in the Wujiang River drainage (Li et al. 1996), while *S. xichouensis*, *S. yimenensis* and *S. wenshanensis* occur in the Red River drainage (Li et al. 2005; Pan et al. 2013; Yang et al. 2017). *Sinocyclocheilus* species are typically found in pools and subterranean streams within karst caves, or in lakes and rivers that are connected to these underground waterways. Previous molecular phylogenetic analyses indicated that this genus comprises five major clades, of which a species group from northeastern Guangxi, called *S. jii* group, diverged early from the others (Xiao et al. 2005; Zhao YH and Zhang 2009; Mao et al. 2022; Wen et al. 2022).

In this study, we sequenced, assembled and annotated the complete mitochondrial genomes of *Sinocyclocheilus guilinensis* Ji, 1985 and *Sinocyclocheilus huangtianensis* Zhu et al. 2011 and analyzed their phylogenetic position within *Sinocyclocheilus* in comparison with published genomes of other *Sinocyclocheilus* species. The mitochondrial genomes determined in this study serve as a valuable addition to existing data for phylogenetic research on the *Sinocyclocheilus* genus.

## Materials and methods

**Figure 1.**
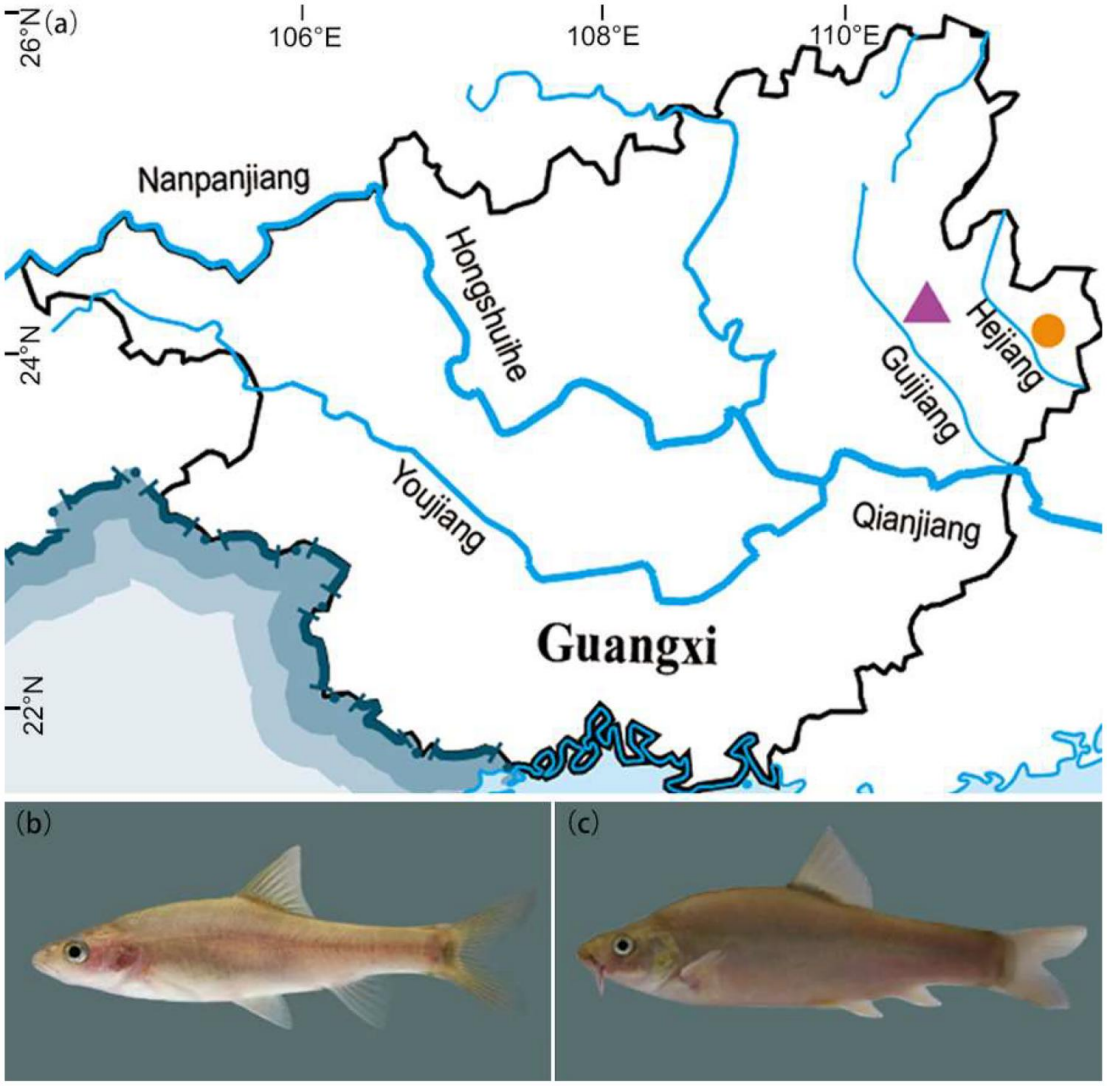
Locality of species of the *Sinocyclocheilus guilinensis* and *S. huangtianensis* in China (a) (purple triangle for *S. guilinensis*, orange circle for *S. huangtianensis*) and photograph of and *S. guilinensis* (b) and *S. huangtianensis*, and (c) (by You He).

The sample of *S. guilinensis* was collected from the Guijiang River, Guilin City, Guangxi Zhuang autonomous region, China (24°39′N, 110°28′E) (Figure 1b). The sample of *S. huangtianensis* was collected from a cave of the Hejiang River, Hezhou City, Guangxi Zhuang autonomous region, China (24°27′02″N, 111°31′30″E) (Figure 1c). The distribution of these species is shown in Figure 1a. The fish was euthanized with ms222 before fin clips being taken. The specimen were fixed with 95% ethanol and preserved in Shanghai Ocean University, China. (sample ID: gui201606 and R2018040401, contact person: Dr Ya Zhang, email: zhangya@shou.edu.cn). We strictly follow the ARRIVE guidelines and have obtained the approval of the Ethics Committee of Shanghai Ocean University for the laboratory animals used in this study.

Total genomic DNA was extracted using the Ezup Column Animal Genomic DNA Extraction Kit (B518251; Sangon Biotech, Shanghai, China) following the manufacturer’s instructions. A second-generation sequencing library was constructed for each sample with a starting DNA material of 300–1000 ng according to Meyer and Kircher (2010) and Li et al. (2013). Briefly, the DNA samples were sheared to approximately 250 bp using a Covaris M220 ultrasonicator (Covaris, Inc., MA, USA). The fragmented DNA samples were checked using electrophoresis with a 1.5% agarose gel. The library construction process include end repair, ligation and fillin (Meyer and Kircher 2010; Li et al. 2013). After amplification, the library products of all samples were mixed in equimolar, and DNA fragments larger than 1 kb were removed by gel extraction. The recovered samples were sequenced on an Illumina HiSeq platform with 2 × 150 paired-end configuration at Genewiz (Suzhou, China).

The raw data for *S. guilinensis* and *S. huangtianensis* were trimmed using Trim_galore (v 0.6.7) and Cutadapt (v1.2.1) to remove the adaptor sequences and low-quality reads (FastQC < 20). The cleaned data of each sample was assembled using Mito Z v2.4 (Meng et al. 2019) with default parameters for annotation and visualization. Seventeen mitochondrial genomes were downloaded from NCBI, including 14 species of *Sinocyclocheilus* and three outgroups (*S. jii* NC_037197 belongs to *the S. jii* group; *S. microphthalmus* MN145877 and *S. anshuiensis* KR069120 belong to *the S. microphthalmus* group; *S. angularis* NC_057312, *S. altishoulderus* NC_013186, *S. bicornutus* NC_031382 and *S. rhinocerous* KR069119 belong to the *S. angularis* group; *S. cyphotergous* NC_072977, *S. multipunctatus* MG026730 and *S. longibarbatus* NC_056194.1 belong to *the S. cyphotergous* group; *S. grahami* NC_013189, *S. wenshanensis* MW553076, *S. tingi* NC_039594 and *S. anophthalmus* NC_023472 belong to *the S. tingi* group; and *Carassius auratus*, KJ874430; *Cyprinus carpio*, OL699932; *Barbus barbus*, NC_008654). The 13 protien-coding sequences of the mitochondrial genome were extracted for phylogenetic reconstruction. Multiple sequence alignment was conducted using MAFFT (Katoh and Standley 2013), and the phylogenetic tree was reconstructed using MEGA11 (Tamura et al. 2021) based on the maximum likelihood method with a bootstrap value of 1000.

## Results

**Figure 2.**
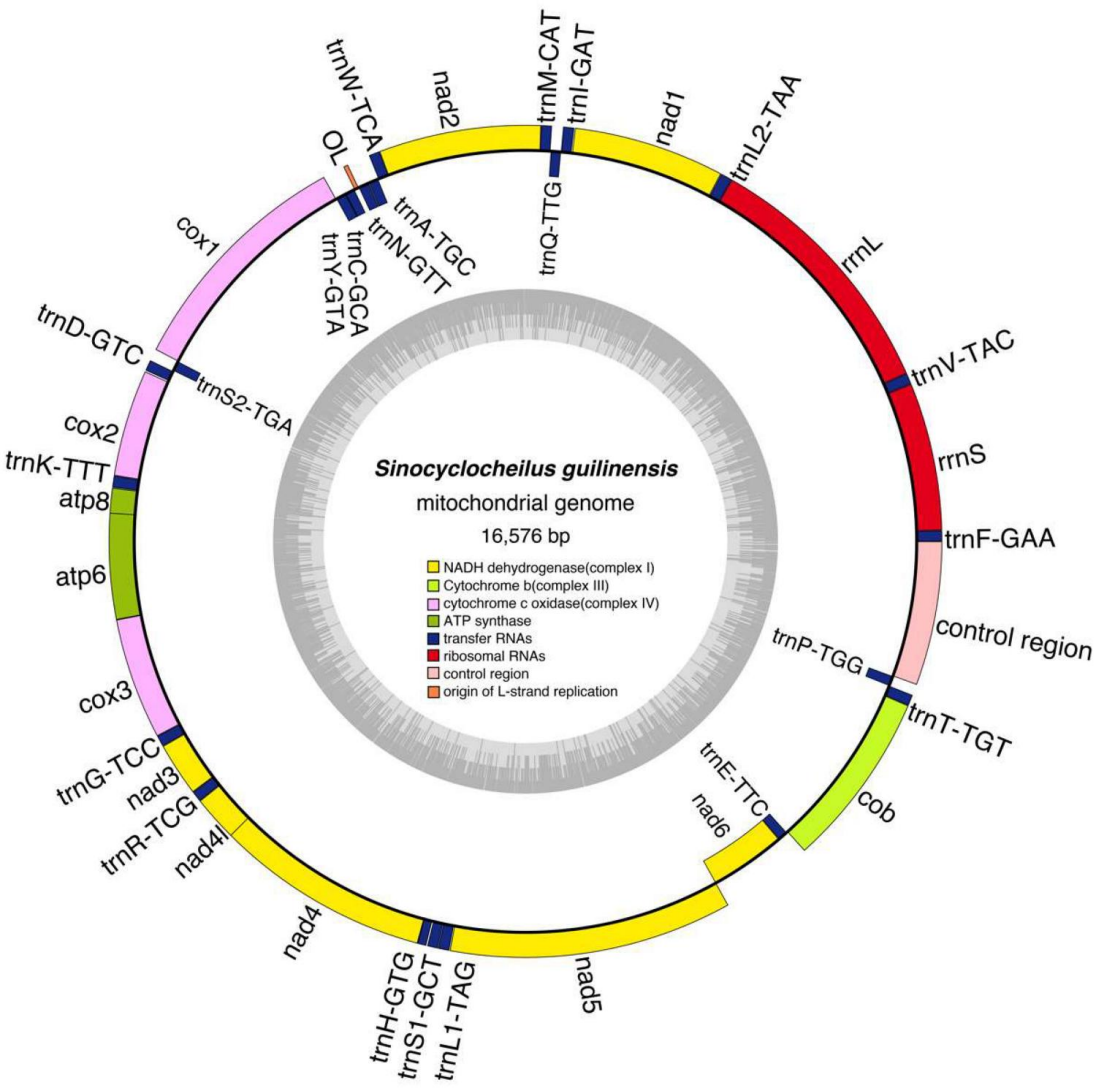
Gene maps of the mitochondrial genome of *Sinocyclocheilus guilinensis.*

**Figure 3.**
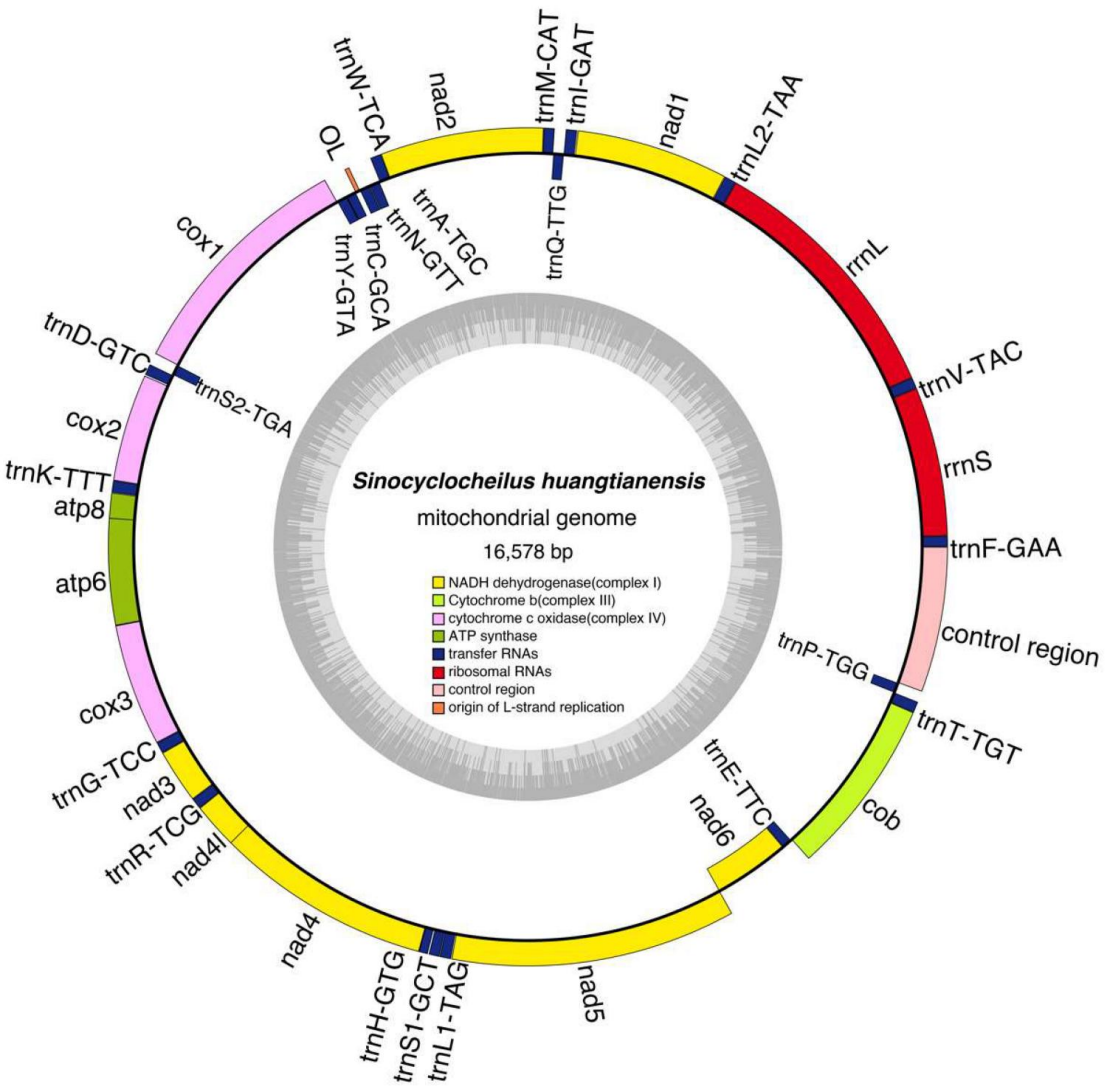
Gene maps of the mitochondrial genome of *Sinocyclocheilus huangtianensis.*

**Figure 4.**
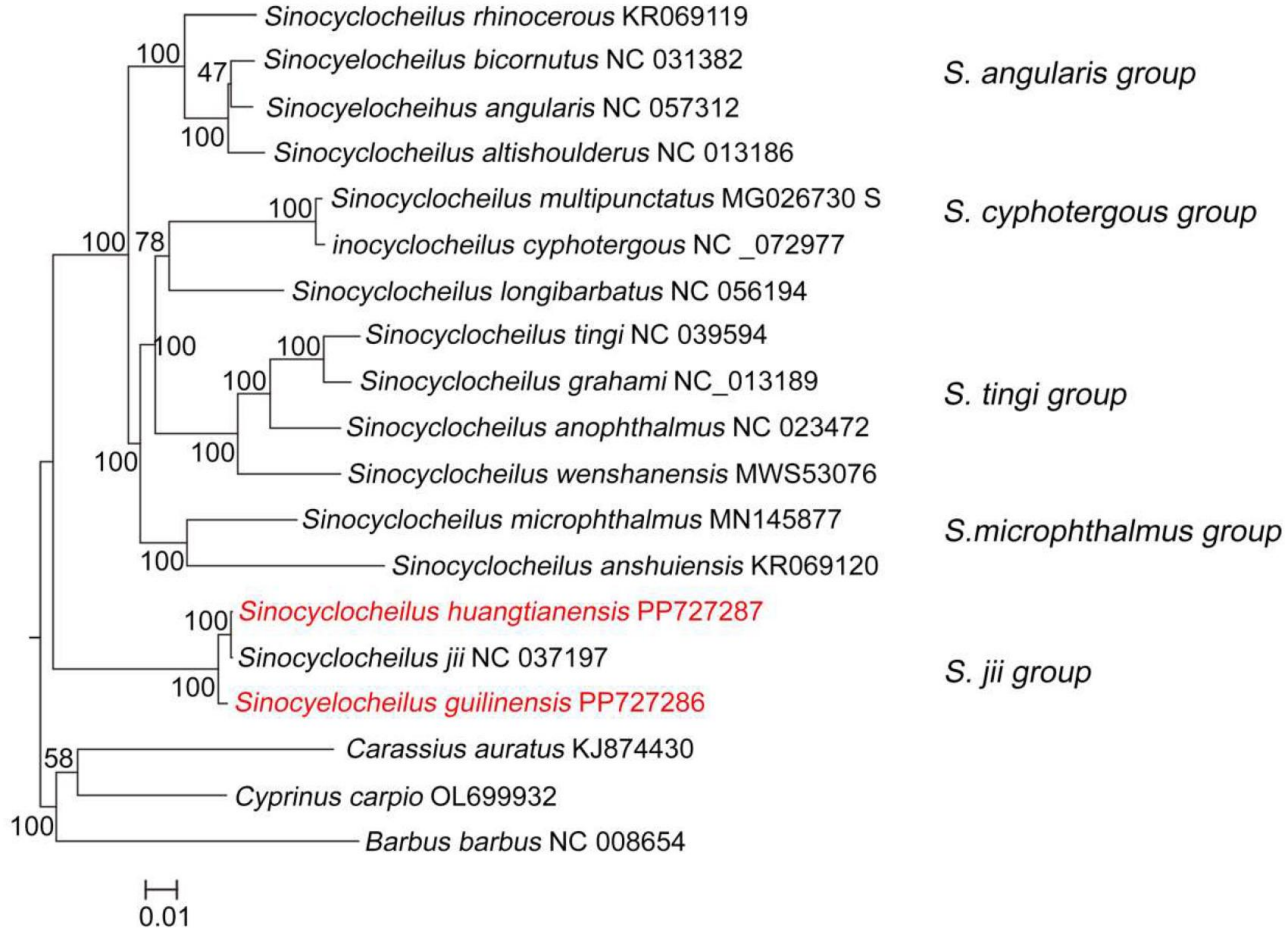
A maximum likelihood tree based on complete mitogenomes of *Sinocyclocheilus guilinensis* (PP727286) and *S. huangtianensis* (PP727287) and 14 other species of the genus *Sinocyclocheilus*. The following sequence were used: *S. jii* NC_ 037197 (Li et al. 2017), *S. microphthalmus* MN145877 (Li P and Yang 2019), *S. anshuiensis* KR069120 (He et al. 2015, unpublished), *S. angularis* NC_057312 (Luo and Zhao, unpublished), *S. altishoulderus* NC_013186 (Wu et al. 2010), *S. bicornutus* NC_031382, *S. rhinocerous* KR069119, *S. cyphotergous* NC_072977, *S. multiunctatus* MG026730 (Zhang and Wang 2018), *S. longibarbatus* NC_056194, *S. grahami* NC_013189 (Wu et al. 2010), *S. wenshanensis* MW553076 (Cui et al. 2021, unpublished), *S. tingi* NC_039594 (Li et al. 2019), *S. anophthalmus* NC_023472, *Carassius auratus* KJ874430, *Cyprinus carpio* OL699932, *Barbus barbus* NC_008654 (Saitoh et al. 2006).

The raw resequencing data of *S. guilinensis* and *S. huangtianensis* were 1.82 G and 2.82 G. The complete mitochondrial genome sequences and annotation files for these species have been uploaded to Genbank under accession number PP727286 and PP727287. The length of assembled mitochondrial genomes regions of *S. guilinensis* and *S. huangtianensis* were 16,576 bp and 16,578 bp respectively and the D-loop region were 929 bp and 931 bp respectively (Figures 2 and 3), Genome coverage and depth were detailed in figure S1 and S2. The mitochondrial genomes of *S. guilinensis* and *S. huangtianensis* each contained 37 genes, including 13 protein-coding genes (PCGs), 22 transfer RNAs (tRNAs), 2 ribosomal RNA genes (rRNAs) as well as a control region (D-loop). The gene order of both species is consistent with known mitochondrial genomes of fish species of the genus *Sinocyclocheilus* (Luo et al. 2021; Zhao et al. 2023). The base content of *S. guilinensis* was 31.83% A, 24.94% T, 16.17% G, and 27.06% C, whereas those of *S. huangtianensis* was 31.79% A, 25% T, 16.22% G, and 26.99% C. Among these genes, only the COI gene has a start codon of GTG, while all other genes have a start codon of ATG. All 13 protein-coding genes are terminated by the termination codon TAA, TAG, or a single T base. The phylogenetic tree constructed based on the maximum likelihood method is shown in Figure 3. Five groups were recognized, *S. jii*, *S. microphthalmus*, *S. tingi*, *S. cyphotergous* and *S. angularis* with the *S. jii* group as the earliest branching clade *Sinocyclocheilus huangtianensis* was sister to *S. jii*, and then grouped with *S. guilinensis* (Figure 4).

## Discussion and conclusion

The phylogenetic analyses of the mitochondrial genomic sequences from 16 species suggested that genus *Sinocyclocheilus* should be a monophyletic group, which was divided into five clades. These clades correspond to the previously delineated groups, namely, *S. jii* group, *S. angularis* group, *S. microphthalmus* group, *S.* c*yphotergous* group and *S. tingi* group (Xiao et al. 2005; Zhao and Zhang, 2009; Wen et al. 2022; Mao et al. 2022; Jiang et al. 2023). Both *S. guilinensis* and *S. huangtianensis* belonged to the *S. jii* group, with *S. guilinensis* was the earliest branch diverged from the *S. jii* group, and *S. huangtianensis* and *S. jii* being mutually sister clades. Geological events have been significant drivers of species diversification (Parmesan and Yohe 2003; Gillespie and Roderick 2014). The uplift of the Yunnan-Guizhou Plateau and the Qinghai-Tibetan Plateau, which shared a common geological history, which led to the formation and uplift of the Zhujiang drainage, the Nanpanjiang drainage, the Beipanjiang drainage, the Hongshuihe drainage and the Youjiang drainage. These geologic events and the changes in the water systems may have led to the differentiation of the species of *Sinocyclocheilus*. (Clark et al. 2004; Xiao et al. 2005; Zhao YH and Zhang 2009).

## Supplementary Material

Supplementary Material.docx

## Data Availability

The genome sequence data that support the findings of this study are openly available in the GenBank of NCBI at https://www.ncbi.nlm.nih.gov under the accession number are PP727286 and PP727287. The associated BioProject, SRA, and Bio-Sample numbers are: PRJNA1112466, SRR29060807, SRR29060806, SAMN41427846 and SAMN41427847, respectively.

## Vulnerable species statement

We have strictly followed the policies of the International Union for Conservation of Nature (IUCN) policies research involving species at risk of extinction, the Convention on Biological Diversity and the Convention on the Trade in Endangered Species of Wild Fauna and Flora.
