# Supplementary material for: The complete mitochondrial genomes and phylogenetic analysis of two Chinese endemic cave fishes, Sinocyclocheilus guilinensis and S. huangtianensis (Cypriniformes: Cyprinidae)
Source: Mitochondrial DNA B Resour. 2025 Feb 2;10(3):167–72. doi: 10.1080/23802359.2025.2460776 (PMC11792122; doi:10.1080/23802359.2025.2460776)
Supplement: Supplementary Material.docx [file TMDN_A_2460776_SM6814.docx]

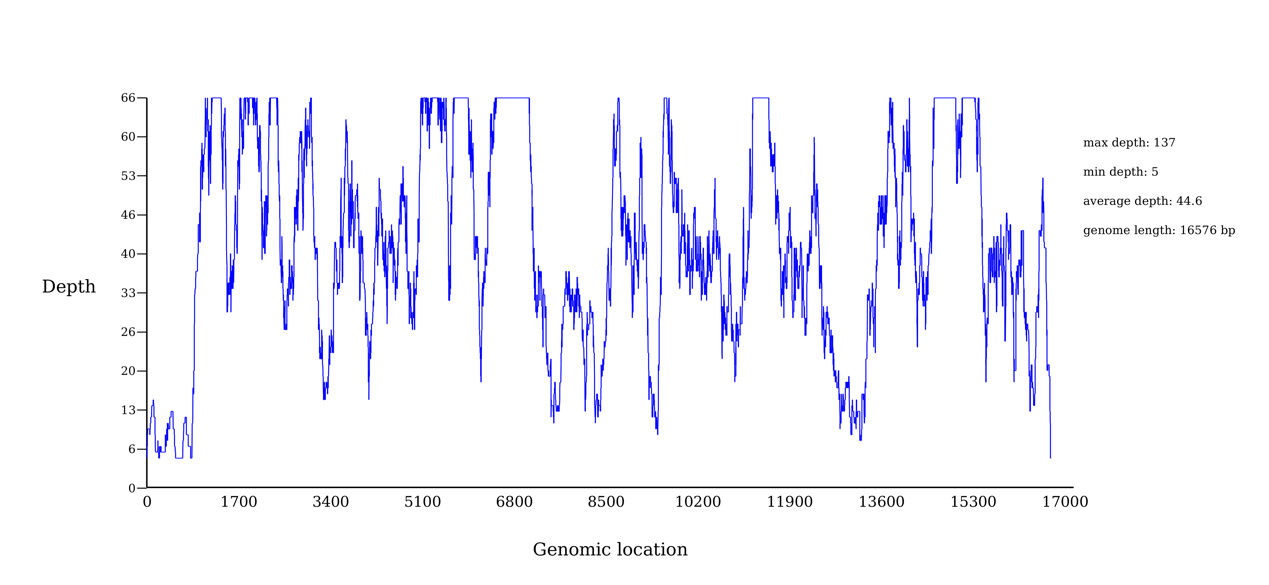


Figure1 Depth map of the mitochondrial genome of *Sinocyclocheilus guilinensis*


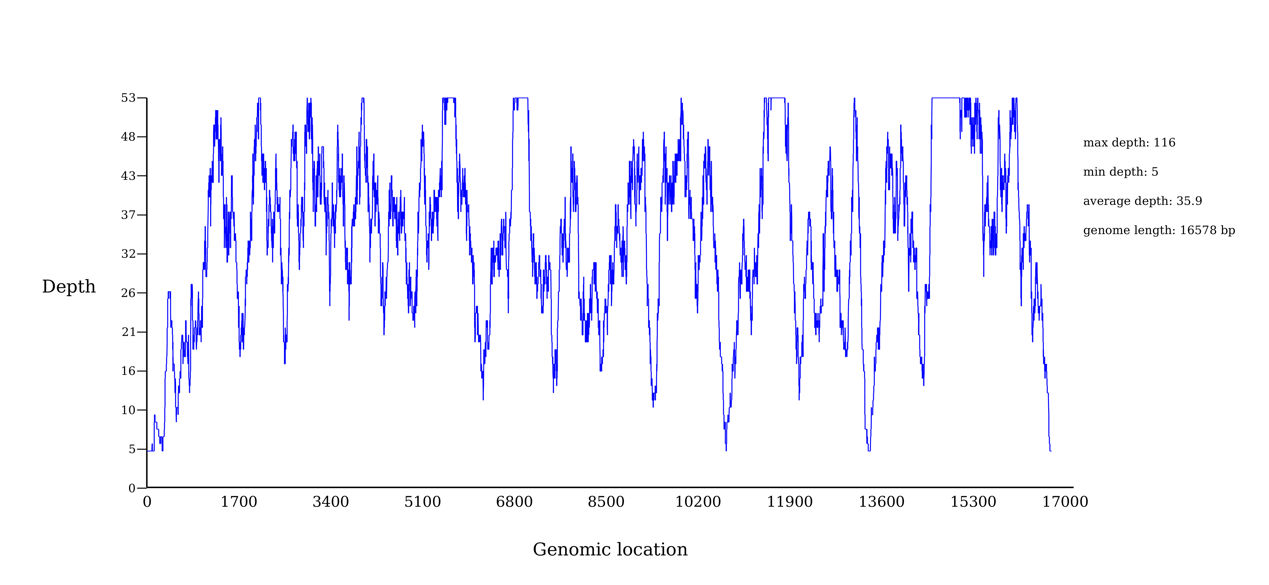


Figure2 Depth map of the mitochondrial genome of *Sinocyclocheilus huangtianensis*
